# Differential physiological responses and tolerance to potentially toxic elements in biodiesel tree *Jatropha curcas*

**DOI:** 10.1038/s41598-018-20188-5

**Published:** 2018-01-26

**Authors:** Minami Yamada, Goitseone Malambane, Satoshi Yamada, Sony Suharsono, Hisashi Tsujimoto, Baleseng Moseki, Kinya Akashi

**Affiliations:** 1 Graduate School of Agriculture Study, Tottori University, 4-101 Koyama-Minami, Tottori, 680-8553 Japan; 2 The United Graduate School of Agricultural Sciences, Tottori University, 4-101, Koyama-minami, Tottori, 680-8553 Japan; 3 Research Center for Bioresources and Biotechnology, Bogor Agricultural University, Gd. PAU, Kampus IPB Darmaga, Bogor, 16680 Indonesia; 4 Arid Land Research Center, Tottori University, 1390 Hamasaka, Tottori, 680-0001 Japan; 5 Department of Biological Sciences, University of Botswana, Private Bag UB, 00704 Gaborone, Botswana

## Abstract

Environmental pollution by potentially toxic elements (PTEs) has become a serious problem with increasing industrialization and the disturbance of natural biogeochemical cycles. Jatropha is an oilseed-bearing shrub with high potential for biodiesel production in arid regions. In this study, we examined the physiological responses of this plant to five representative PTEs (Cd, Cr, Cu, Ni, and Zn) in a hydroponic culture. Application of higher concentrations of Cd and Zn led to severe leaf chlorosis, and Cd, Cu, and Ni treatments resulted in significant growth retardation. Higher enrichment of the applied PTEs in the shoots was observed for Zn- and Cd-treated plants, with the latter reaching 24-fold enrichment in plants exposed to 10 μM Cd, suggesting that Jatropha can cope with relatively higher internal concentrations of toxic Cd. Although Cd stress led to the disturbance of essential mineral homeostasis and photosynthesis, this induced an increase in thiol compounds in the roots, suggesting defensive responses of Jatropha to PTEs. This study showed that Jatropha exhibits distinct sensitivities and physiological responses to different PTEs. This study also provides basic knowledge for diagnosing the physiological status of Jatropha trees for potential dual use in afforestation and as a sustainable energy supply.

## Introduction

Pollution by potentially toxic elements (PTEs), which adversely affect ecosystems, is a global issue jeopardizing environmental sustainability^1^. This pollution is largely due to anthropogenic activities, including mining, dredging and civil engineering, industrial discharge, and overuse of agrochemicals. Although some PTEs, such as chromium (Cr), copper (Cu), manganese (Mn), nickel (Ni), and zinc (Zn), are essential for plant growth, at high concentrations, they are known to be toxic^2^. Other PTEs, such as cadmium (Cd), which is dispersed as waste material from the mining industry and in sludge-amended and Cd-rich phosphatic fertilized soils^3^, are normally not regarded as nutrients. Cd contamination in soil and water often adversely affects agricultural production and food security in downstream regions^4^.

Jatropha (*Jatropha curcas* L.) is a deciduous shrub of the family *Euphorbiaceae*, and is capable of producing high levels of triacylglycerol for biodiesel feedstock^5^. Jatropha is tolerant to water deficit conditions; therefore, it has attracted attention as a viable next-generation biodiesel feedstock in arid regions. The tolerance of Jatropha to soils contaminated with PTEs was recently reported, and this has drawn considerable attention to the potential utility of Jatropha for biomass fuel production on polluted land^6–8^. Although Jatropha has not been regarded as hyperaccumulator of PTEs^9^, PTEs-tolerant plant species with economically beneficial traits, such as feedstock for biofuel, may have potential advantages for sustainable economic development and afforestation in PTE-contaminated areas^10^. However, detailed physiological analyses of this plant with regard to different PTEs are currently limited. In the field, PTEs have a complex interaction with soil particles, which often complicates the generalization of knowledge on physiological responses to each PTE. On the other hand, hydroponic culture enables direct measurements of plant physiological parameters and metabolism in a time-dependent manner, and this method is widely used to determine the phytotoxic effects by PTEs^11^. Acute responses to PTEs are informative indicators for surveying stress resistance and underlying molecular mechanisms^12^. In the preset study, therefore, we examined various physiological responses of Jatropha to 12-day exposure to five representative PTEs (Cd, Cr, Cu, Ni, and Zn) using a hydroponic cultivation system.

## Results

### Leaf phenotype after exposure to PTEs

**Figure 1:**
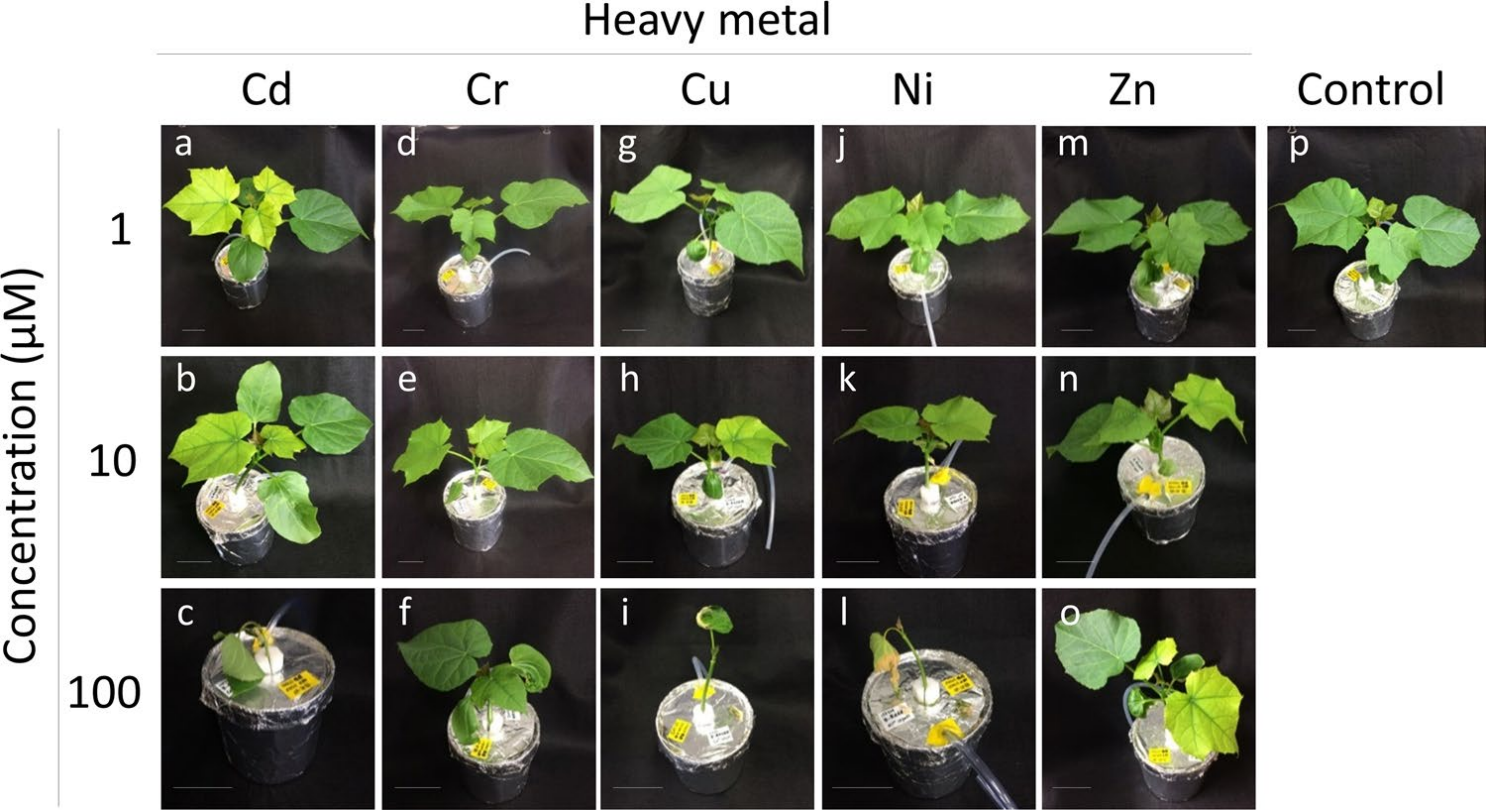
Phenotype of Jatropha after exposure to PTEs. Jatropha seedlings were grown in half-strength Hoagland’s solution until their first leaf was fully developed, then CdSO_4_ (**a**–**c**), Cr_2_(SO_4_)_3_ (**d**–**f**), CuSO_4_ (**g**–**i**), NiSO_4_ (**j**–**l**), and ZnSO_4_ (**m**–**o**) were added at a final metal concentration of 1 µM (**a**,**d**,**g**,**j**,**m**), 10 µM (**b**,**e**,**h**,**k**,**n**), and 100 µM (**c**,**f**,**i**,**l**,**o**). Plants without the addition of PTEs were used as a control (**p**). The plants were photographed after 12 days of exposure to the PTEs. Scale bar = 5 cm.

Germinated Jatropha seedlings were grown in half-strength Hoagland solution until their first leaf was fully developed, and then solutions of five PTEs (Cd, Cr, Cu, Ni, and Zn) were added to the hydroponic solution at final metal concentrations of 1, 10, and 100 µM. At 12 days after the onset of PTE treatment, Jatropha plants exposed to 1 µM Cd showed chlorosis symptoms on newly expanded second leaves, suggesting a decrease in chlorophyll content (Fig. 1a). The phenotypes of Jatropha plants exposed to 1 µM Cr, Cu, Ni, and Zn were essentially similar to those of the control plants (Fig. 1d,g,j,m,p). At a metal concentration of 10 µM, chlorosis in the second leaf was observed not only in Cd-treated plants but also in Cu- and Zn-treated plants (Fig. 1b,h,n). At the highest metal concentration of 100 µM, plant growth was strongly inhibited, especially in plants treated with Cd, Cu, or Ni, which had withered and fallen leaves (Fig. 1c,i,l). Although leaf withering was not observed in plants treated with 100 µM Cr or Zn, growth retardation was evident under these conditions (Fig. 1f,o).

**Figure 2:**
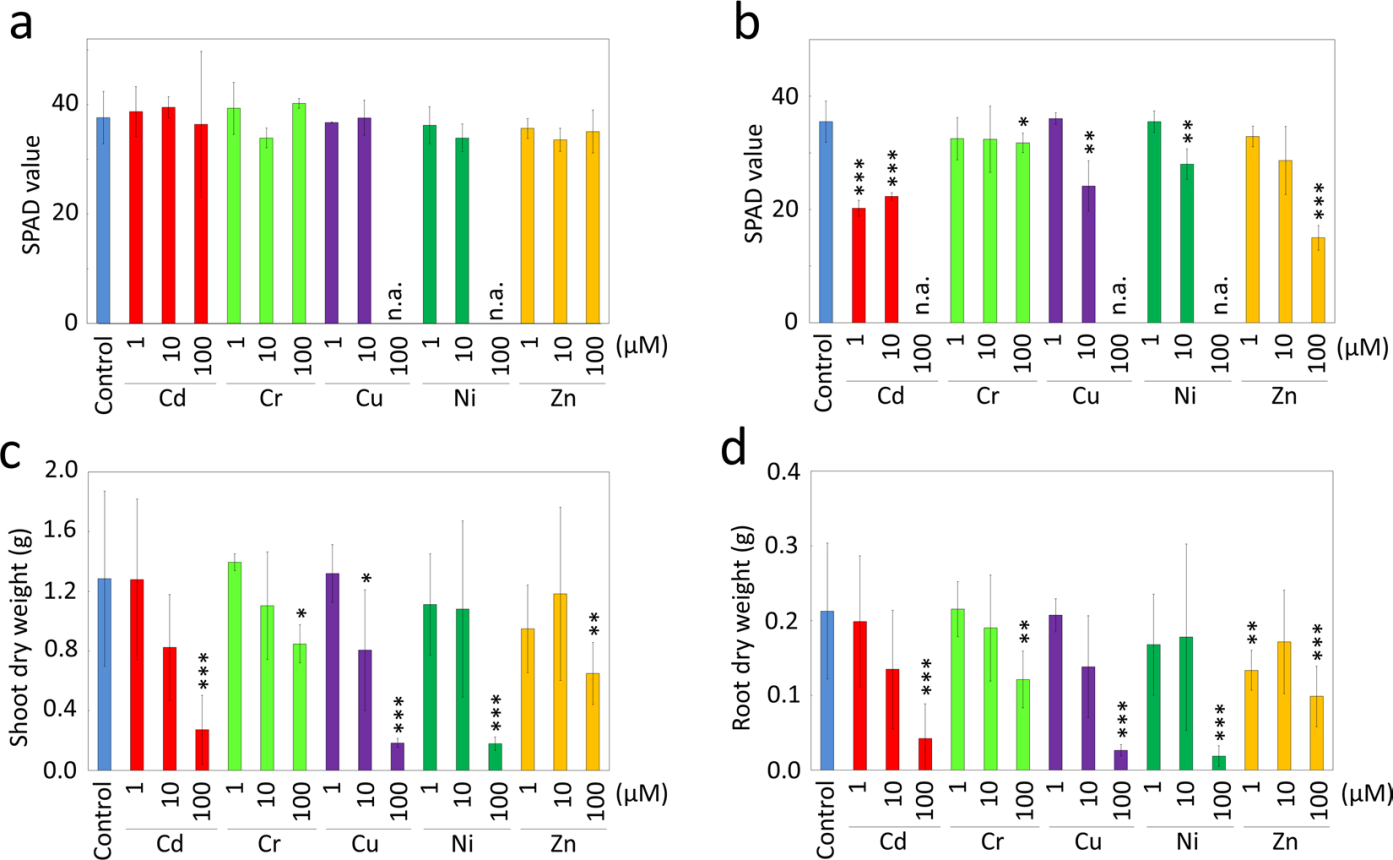
Jatropha growth after exposure to PTEs. SPAD values, an index of chlorophyll content, were measured for the first (**a**) and second (**b**) leaves 12 days after applying the indicated concentration of the PTEs. Jatropha plants were then harvested, and the dry weights of shoot (**c**) and root (**d**) tissues were measured. Data are the average ± SD of three individuals. n.a., not analyzed because the leaves had died. Statistical differences between the PTE-treated and untreated control plants were tested using a *t*-test. ***1%; **5%; *10%.

Consistent with these visual observations, a significant decrease in the SPAD value, an index of chlorophyll content in the leaves^13^, was observed in the second leaves of plants treated with 1 and 10 µM Cd, 10 µM Cu, and 100 µM Zn (Fig. 2b). The SPAD values of the plants treated with 100 µM Cd, Cu, and Ni were not measured because these leaves had withered and fallen. In contrast, the first true leaf, which had already fully developed before the addition of metals, remained green (Fig. 1), and SPAD values were unchanged except in plants treated with 100 µM Cu and Ni (Fig. 2a), in which the first true leaves had fallen (Fig. 1i,l).

### Roots phenotype after exposure to PTEs

**Figure 3:**
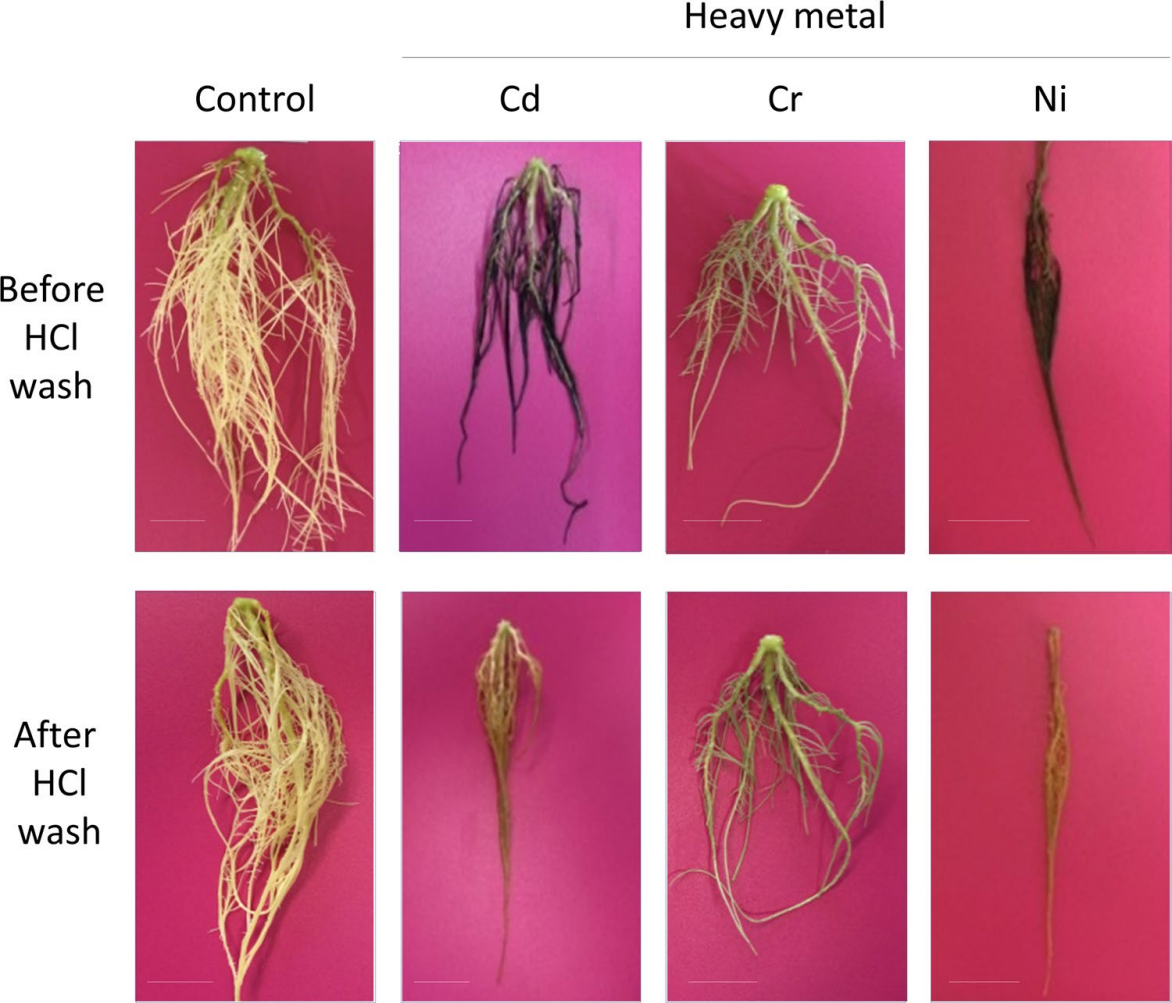
Colorimetric changes of Jatropha roots after exposure to PTEs. Jatropha plants were grown in hydroponic culture in the presence of Cd, Cr, or Ni metal ions at a concentration of 100 µM for 12 days, and the appearance of the roots was photographed immediately after harvesting for Cd (**c**), Cr (**e**), and Ni (**g**) treatments. Subsequently, the harvested roots were washed with 1 M HCl solution for 1 min, followed by a solution of 0.1 M EDTA, and then rinsed with distilled water and re-photographed (**d**,**f**,**h** for Cd, Cr, and Ni treatments, respectively). Roots of control plants before and after the same washing procedure are presented in (**a**) and (**b**), respectively. Scale bar = 3 cm.

Observations on the roots of Jatropha plants in the control condition showed a whitish-beige surface color, which did not significantly change throughout the cultivation period (Fig. 3a). Treatment with 100 µM Cd, Cr, and Ni resulted in a significant change in the color of the roots. Roots cultivated in the presence of 100 µM Cd or Ni turned black (Fig. 3c,g), whereas Jatropha roots cultivated with 100 µM Cr became pale green in color (Fig. 3e). No color changes were observed in roots cultivated in Cu- and Zn-containing hydroponic solutions (data not shown). Harvested roots were washed in distilled water and then soaked in 1 M HCl solution for 1 min. This treatment removed the black color from the Cd- and Ni-treated roots and resulted in a residual dark-yellowish color (Fig. 3d,h). In contrast, HCl treatment did not remove the greenish component from Cr-treated roots, and they remained essentially the same color (Fig. 3f). The metal contents in the 0.1 M HCl washing solutions were analyzed using ICP-AES. The result showed high concentrations of the applied PTEs in the HCl wash solutions (Supplementary Table S1): 7,129 nmol Cd was recovered from the plants cultivated with Cd, which was 1,398-fold higher than the control conditions. Similar trends were observed in Cr- and Ni-treated conditions: 616- and 199-fold higher concentrations of the applied PTEs were observed in Cr- and Ni-treated plants, respectively, as compared to the control.

### Plant biomass after exposure to PTEs

The biomass of Jatropha plants after treatment with different PTEs was evaluated by examining the dry weight of shoots and roots after the treatment (Fig. 2c,d). At a PTE concentration of 1 µM, the dry weights of shoots and roots treated with each of the five metals were not statistically different from the control, except for a 37% decrease in the dry weight of roots treated with 1 µM Zn (*P* < 0.1). Higher concentrations of the PTEs led to a marked decrease in dry weights of both shoots and roots. In particular, the dry weights of shoots and roots of plants treated with 100 µM Cd, Cu, and Ni were significantly decreased by 80–91% as compared with the control.

### Absorption of PTEs by Jatropha tissues

**Figure 4:**
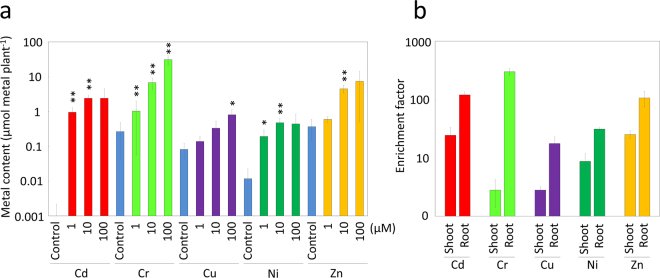
Accumulation of PTEs in Jatropha plants. (**a**) Jatropha plants were harvested 12 days after exposure to different concentrations of PTEs, and the contents of the applied metals that had accumulated in the plants were analyzed. Values are expressed as the metal content per individual plant. Statistical differences between the metal-treated and untreated control plants were tested using a *t*-test. ***1%; **5%; *10%. (**b**) Jatropha plants were exposed to the respective PTEs at a concentration of 10 µM for 12 days, and then the contents of the applied PTEs in the shoots and roots were analyzed separately and expressed as an enrichment factor showing the fold difference in metal concentration between the hydroponic solution and the plant tissues. Data are the average ± SD of three individuals.

Accumulation of the PTEs in the whole plant was measured after the treatment. For every growth condition, the applied PTE itself was markedly accumulated to higher levels as compared with the control plants on an individual plant basis, whereas in each case, the levels of the other four PTEs were either statistically unchanged or affected to a lesser extent (Supplementary Table S2; Fig. 4). In the Cr and Cu treatments, the accumulated PTE increased up to the 100 µM treatment, whereas in the Cd and Ni treatments, the PTE accumulation reached a peak with the 10 µM treatments, and no further increase was observed with the 100 µM treatment (Fig. 4a, Supplementary Table S2). The highest PTE accumulation was observed with the 100 µM Cr treatment, where the value reached 31.5 µmol Cr g^−1^ dry weight (DW), which corresponded to 31.0 µmol Cr plant^−1^. In the 100 µM Zn and Cd treatments, the accumulation levels of applied PTE reached 9.7 µmol Zn g^−1^ DW and 7.6 µmol Cd g^−1^ DW, which corresponded to 7.4 µmol Zn plant^−1^ and 2.4 µmol Cd plant^−1^, respectively.

The potential of Jatropha as a tool for the phytoextraction of PTEs was estimated using enrichment factors (EFs)^14^, which indicate the difference in concentration between an organism and its environment. With PTEs at a concentration of 1 µM in the hydroponic solution, Jatropha plants showed higher EFs in the range of 72–88 for Cd-, Cr-, and Zn-treated plants as compared with a lower range of 11–19 for Cu- and Ni-treated plants (Supplementary Table S3). The EF decreased as metal concentration increased in the hydroponic solution, and decreased to values of 2–13 at 100 µM concentration for most metals except for Cr, which retained a high EF value of 40.6.

To examine the distribution of PTEs in Jatropha, shoot and root tissues of plants subjected to treatment with the respective metals at a concentration of 10 µM were measured separately. Higher metal concentrations were observed in the root tissues, whereas the concentration in the shoots was lower in relative terms for all metal treatments (Supplementary Table S4; Fig. 4b). From the observed metal concentrations and biomass of the shoots and roots, it was estimated that 8.8%, 7.1%, 11.7%, and 10% of Cd, Cu, Ni, and Zn absorbed by the plants were translocated to the shoot tissues, respectively. In contrast, a distinct behavior was observed in Cr-treated plants, in which only 0.5% of the absorbed metal was translocated to the shoot tissues (Supplementary Table S4). In the shoots, the EF values were higher in Cd- and Zn-treated plants (24.4 ± 8.9 and 25.2 ± 3.8, respectively), whereas in the roots, the EF values were the highest in Cr-treated plants and reached 300 ± 40.4 (Fig. 4b).

### Metabolism of essential minerals after exposure to PTEs

**Figure 5:**
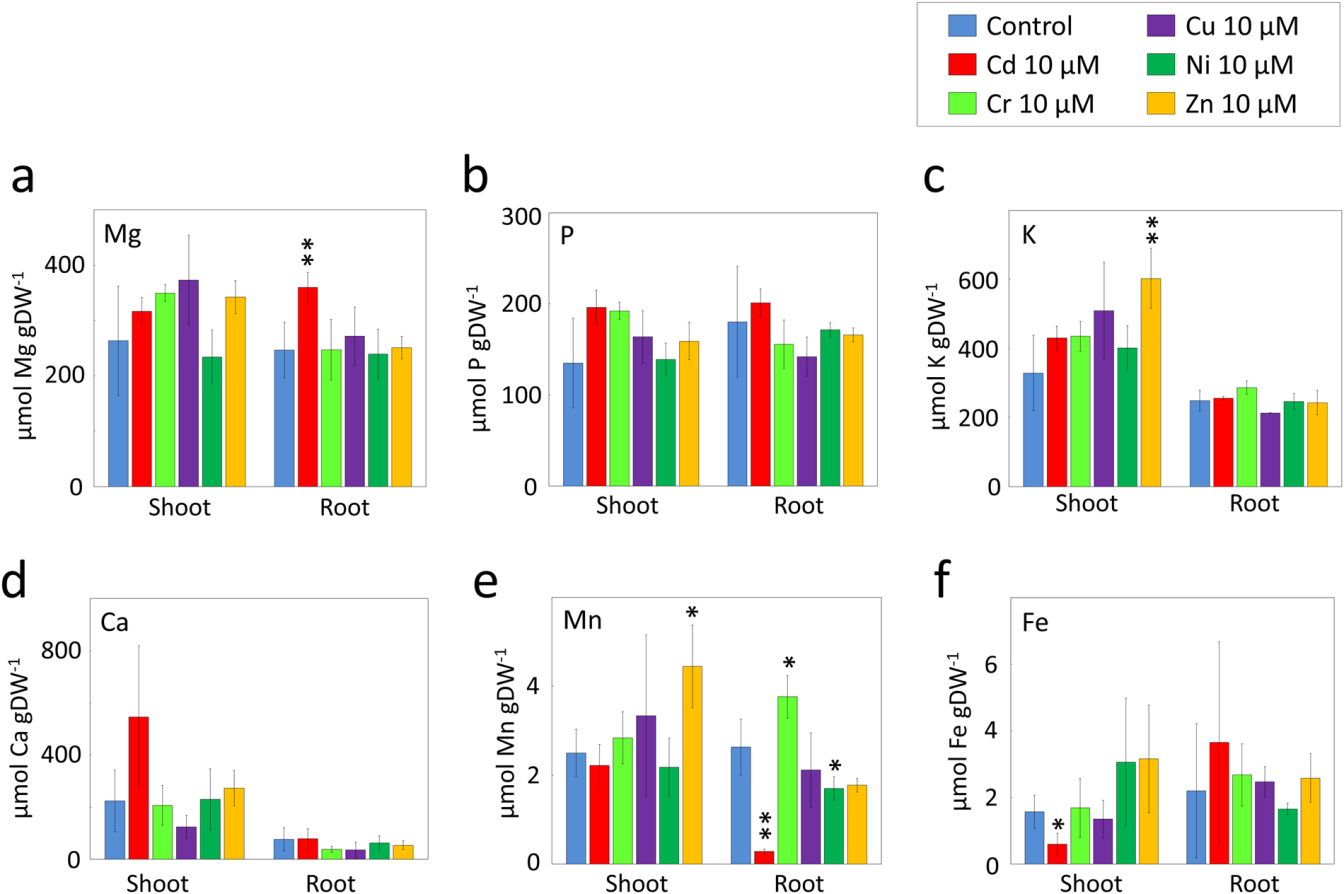
Effects of PTE exposure on the accumulation of essential minerals. Jatropha plants were harvested 12 days after exposure to the respective PTEs at a concentration of 10 µM, and the contents of representative essential mineral nutrients in the shoots and roots were analyzed. The measured essential minerals were Mg (**a**), P (**b**), K (**c**), Ca (**d**), Mn (**e**), and Fe (**f**). Data are the average ± SD of three individuals. Statistical differences between the metal-treated and untreated control plants were tested using a *t*-test. ***1%; **5%; *10%.

The influence of PTE exposure on the tissue content of representative essential mineral nutrients, i.e., magnesium (Mg), phosphorus (P), potassium (K), calcium (Ca), manganese (Mn), and iron (Fe), were analyzed at a PTE concentration of 10 µM in hydroponic solution (Fig. 5). Although the concentrations of these essential mineral nutrients were statistically unchanged under most conditions, a 90% decrease in root Mn content and a 62% decrease in shoot Fe content were observed in Cd-treated plants (Fig. 5e,f). A decrease in root Mn content was also observed in Ni-treated plants (Fig. 5e). On the other hand, increases in essential minerals were observed in some cases; K and Mn contents in shoots increased in Zn-treated plants (Fig. 5c,e), Mg content in roots increased with Cd treatment (Fig. 5a), and Mn content in roots increased with Cr treatment (Fig. 5e). No significant changes were observed in the contents of P or Ca for any of the PTE treatments (Fig. 5b,d).

### Photosynthesis and water relations of leaves after exposure to Cd

**Figure 6:**
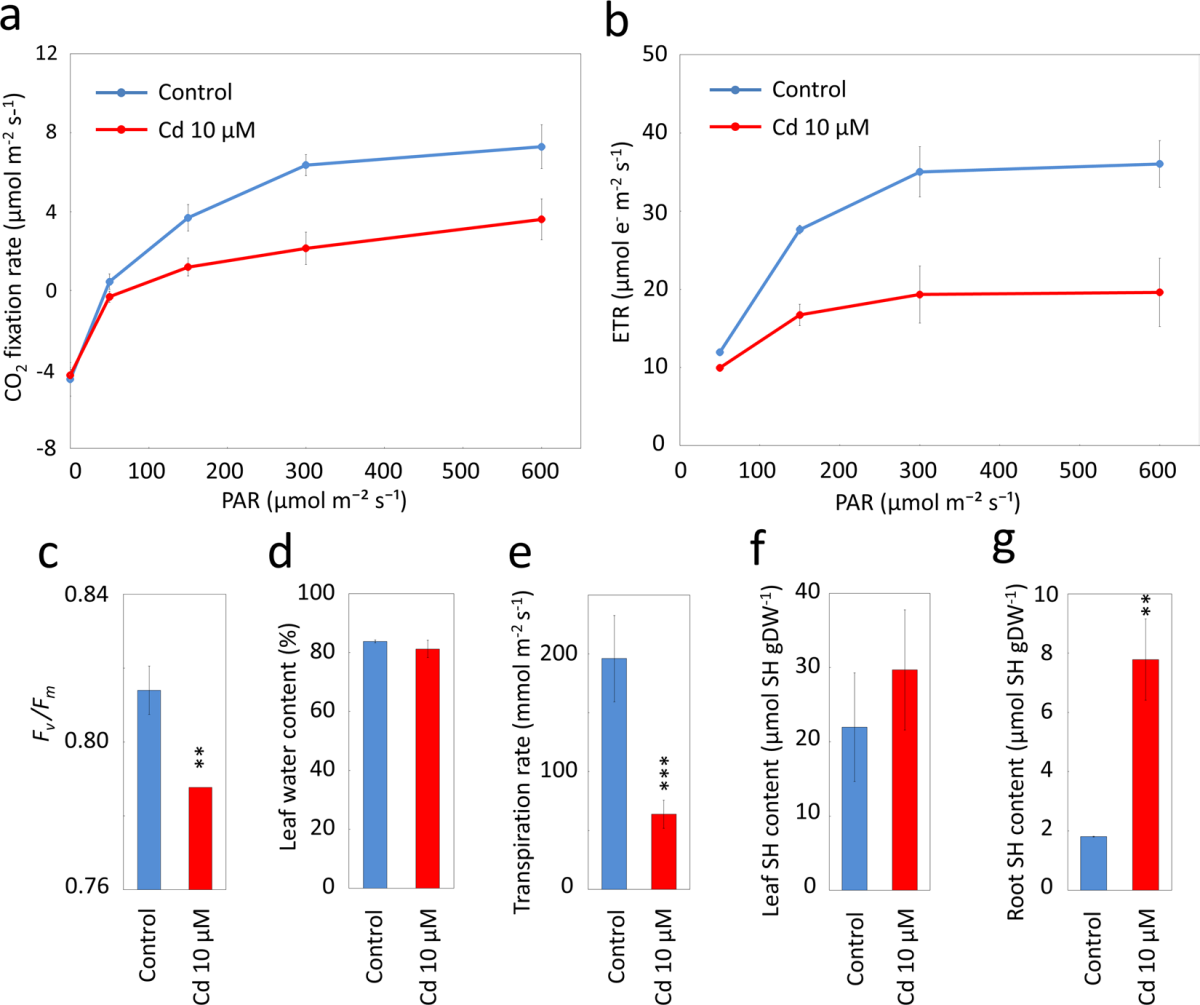
Photosynthesis activity, water relations, and total thiol levels of Jatropha after exposure to Cd. Jatropha plants were exposed to Cd at a concentration of 10 µM for 12 days, and CO_2_ fixation rate (**a**) and electron transfer rate (ETR) through photosystem II (**b**) of their second leaves were measured under different intensities of photosynthetically active radiation (PAR). The maximum quantum yield of photosystem II (**c**), leaf water content (**d**), and transpiration rate (**e**) were analyzed in the second leaves. Accumulation of total thiol compounds in the second leaf (**f**) and roots (**g**) was measured. Data are average ± SD of at least three individuals. Statistical differences were tested using a *t*-test. ***1%; **5%; *10%.

The physiological responses of Cd-treated plants were analyzed in more detail. Net photosynthetic CO_2_ fixation rate and electron transfer rate (ETR) from photosystem II in the second leaves were compared between plants treated with 10 µM Cd and the control. In Cd-treated plants, a 50–68% reduction in CO_2_ fixation rate was observed at an actinic light intensity of 150 µmol m^−2^ s^−1^ and beyond (Fig. 6a). Similar trends were shown for the ETR, for which a 40–46% decrease was observed in Cd-treated plants at an actinic light intensity above 150 µmol m^−2^ s^−1^ (Fig. 6b), showing that electron transfer from PSII was inhibited by Cd treatment. The dark-adapted *F*_*v*_*/F*_*m*_ value, an indicator of photoinhibition^15^, was 0.79 ± 0.0006 in the second leaves of plants after Cd treatment, which was slightly lower than that in control leaves (0.81 ± 0.007) (Fig. 6c), suggesting that photoinhibition in photosystem II caused by Cd treatment in this study was relatively subtle.

Water content in the leaves of plants treated with 10 µM Cd was 81.2 ± 3.0%, which was not statistically different from the value in control plants (83.7 ± 0.5%) (Fig. 6d). In contrast, a significant decrease was observed for the transpiration rate in the leaves of plants treated with Cd (Fig. 6e). The transpiration rate in Cd-treated plants was 63.8 ± 16.0 mmol m^−2^ s^−1^, which was 33% of the value in control plants.

### Total thiol compounds after exposure to Cd

We examined the accumulation level of thiol compounds, which are known protective agents against PTE damage, in the roots and leaves of Jatropha plants exposed to 10 µM Cd treatment. The thiol contents in the Cd-exposed and control leaves were 29.7 ± 8.1 and 22.0 ± 7.3 µmol g^−1^ DW, respectively, but the difference was not statistically significant (Fig. 6f). In contrast, the contents of the thiol compounds in Cd-exposed roots were 7.8 ± 1.4 µmol g^−1^ DW, which was 4.3-fold higher than the value in the control roots (Fig. 6g).

## Discussion

In biological systems, the mechanisms of toxicity of the PTEs have been discussed as either (i) inhibiting essential biological functional groups on biomolecules, (ii) displacing essential metal ions in biomolecules, and/or (iii) disrupting the active conformation of biomolecules^16^. The present study using PTEs at a concentration of 100 µM in hydroponic solution showed that Jatropha dry weight was more susceptible to Cd, Cu, and Ni, whereas Cr and Zn exerted relatively milder effects on dry weight (Fig. 2), suggesting that exposure to different PTEs led to different degrees of growth retardation in Jatropha (Figs 1 and 2). In other photosynthetic organisms, the degree of toxicity of various PTEs, or toxicity sequence, was reported as Hg > Cu > Cd > Fe > Cr > Zn > Ni > Co > Mn for the algae *Chlorella vulgaris*, and as Hg > Pb > Cu > Cd > Cr > Ni > Zn for barley (summarized in^16^). Therefore, the toxicity sequence in Jatropha may share a similar trend to that reported in barley and chlorella. It is noteworthy that Ni treatment resulted in severe growth defects in Jatropha in this study, whereas this metal was reported as a lower rank metal in the toxicity sequence in barley and chlorella. Ni is recognized as a trace element in plants, and it is essential as a cofactor of metabolic enzymes such as urease^17^. However, Ni is also known for its toxicity in various physiological processes, such as in photosynthesis and other cellular metabolic processes^18,19^. Because the impact of Ni toxicity on plant physiology depends on the growth stage, cultivation conditions, and type of Ni administration, the physiological processes of Jatropha after Ni exposure will need to be examined in more detail in future studies.

Exposure to higher concentrations of Cd and Ni resulted in the development of black deposits in Jatropha roots (Fig. 3). Because these black deposits were easily removed by washing in 1 M HCl solution, it is plausible that this deposit adhered to the surface of the root. The washing solution contained the applied PTEs at a high concentration (Supplementary Table S1), suggesting that the black deposit might contain the applied PTEs. Alternatively, the black deposit might represent substances secreted from plant roots, such as phenolic compounds^20^. In contrast, Cr treatment resulted in the deposition of a pale green color in the roots of Jatropha, and this color was not removed by HCl washing (Fig. 3). These observations suggested that the coloring substances were either insoluble in 1 M HCl or they were absorbed rather deeply into the root tissues and were, therefore, unextractable by HCl washing. Because Cr(III) salt has a green color^21^, it is reasonable to postulate that the pale green coloring in the roots may represent the visualization of accumulation of Cr absorbed in the root tissues. Further research will be needed to examine the possible relationships between the green coloring and Cr accumulation in Jatropha roots.

Among the five PTEs examined in this study, Cr caused lower growth inhibition and was most effectively accumulated in Jatropha (Figs 2 and 4; Supplementary Table S2–S4). However, the shoot/root ratio of Cr accumulation was the lowest among the five PTEs, suggesting that although absorption of Cr by Jatropha roots was efficient, translocation of Cr to the shoot might be blocked in the root tissues. It is noteworthy that the shoot enrichment factor for Cd reached 24.4 ± 8.9, showing the second highest value after Zn (Fig. 4b). Cd is recognized as a very potent pollutant because it is highly toxic and shows high solubility in water^3,4^. In plants, Cd accumulation leads to chlorosis, leaf wilt, and stunting^22^, and it inhibits the absorption, transport, and use of essential nutrients and water^23^. On a dry weight basis, the Cd accumulation level in Jatropha in this study reached 313.2 mg kg^−1^ DW (Fig. 4a; Supplementary Table S2), which was lower than the reported values from hyperaccumulators such as *Polygonum thunbergii* (3,800 mg kg^−1^ DW)^24^ and *Thlaspi caerulescens* (1,800 mg kg^−1^ DW)^25^, suggesting that Jatropha may not be categorized as a Cd hyperaccumulator. However, the value in Jatropha was markedly higher than the reported values in other non-hyperaccumulator trees such as *Salix* sp. (8.86 mg kg^−1^ DW)^26^ and *Acer pseudoplatanus* L. (0.5 mg kg^−1^ DW)^27^, which supports the observation of efficient field performance of Jatropha on Cd-affected land^7^.

It has been reported that the absorption, translocation, and distribution of PTEs at least partially share molecular machinery with essential mineral nutrients in plants^28^. Thus, it is reasonable to suggest that exposure to high concentrations of PTEs may disturb the metabolism of other mineral nutrients in plants^29,30^. In this study, two notable abnormalities in the abundance of essential mineral nutrients were found after Cd exposure: a decrease in Mn content in the roots and in Fe content in the shoots (Fig. 5e,f). Fe is an essential element in plants and has diverse roles in cellular metabolism and defense: it is a cofactor for cytochrome, peroxidase, and catalase, and is also involved in chlorophyll synthesis in plants^31,32^. Chlorosis of Jatropha exposed to the relatively lower Cd concentration observed in this study might reflect disturbance of energy metabolism, antioxidative defense, or chlorophyll synthesis caused by the inhibition of Fe metabolism. Preferential decreases in shoot Fe content might reflect a Cd-triggered block in Fe loading into the xylem element during translocation to the shoots^33^. Mn is an essential component of many metabolic enzymes and plays a pivotal role in water oxidation in photosynthesis^34^. The preferential decrease in root Mn content in Cd-treated Jatropha plants is intriguing. In barley, a root plasma membrane transporter specific for the uptake of Mn and Cd has been characterized^35^, implying that the decrease in root Mn content in Cd-treated Jatropha may reflect competition for uptake of these two metals in Jatropha roots.

In Jatropha, Cd treatment led to a decrease of chlorophyll content in newly developed leaves, together with a reduction in CO_2_ fixation and transpiration rates (Figs 2 and 6). Negative effects of Cd on various stages of photosynthesis and water relations in plants have been well documented^36–38^. In this study, Cd reduced the leaf transpiration rate but not leaf water content in Jatropha (Fig. 6d,e), suggesting that Cd treatment affected stomatal behavior, as has been observed in other plants^38,39^. Thiol compounds were significantly induced in the roots of Cd-exposed Jatropha (Fig. 6g). Previous studies demonstrated that thiol-rich low molecular weight compounds, such as glutathione, metallothionein, and phytochelatin, were induced in Cd-exposed plants and engaged in tolerance by binding Cd^22,40–42^. This observation in Jatropha suggested that although their molecular identity awaits further investigation, these thiol compounds may be induced as defense agents against Cd. It is noteworthy that a significant induction of thiol compounds occurred in the roots but not in the leaves (Fig. 6f,g), which was in accordance with the higher accumulation of Cd in roots as compared with shoots (Fig. 4b)

This study offers new knowledge on the effects of different PTEs on morphology, growth, and various metabolisms in the biodiesel plant Jatropha. The knowledge on the behavior of PTEs and their impact on Jatropha physiology should be useful for the diagnosis of Jatropha performance on PTE-polluted land^6,7^ for the multiple purposes of renewable energy production, phytoremediation and afforestation. In Jatropha cultivation management, extensive pruning of aboveground trunks and branches on an annual basis is a common practice^43,44^. The pruning produces a large amount of non-oil biomass, which may potentially serve as an effective measure for extracting PTEs from contaminated land in addition to producing renewable biodiesel energy. Moreover, substantial variation in agronomic traits and underlying genetic polymorphism has been observed in Jatropha accessions worldwide^5^, emphasizing the need to further evaluate the afforestation and phytoremediation capacities of different Jatropha accessions as well as to breed improved Jatropha varieties for growth on contaminated lands.

## Methods

### Plant material and cultivation conditions

Seeds of Jatropha (*Jatropha curcas* L.) variety IP-3P obtained from The Plantation Research and Development Center (Pakuwon), Ministry of Agriculture, Indonesia were used in this study^45^. Jatropha seeds were planted in a calcined granular diatomaceous earth (Isolite CG No. 2, Isolite Insulating Products, Osaka, Japan), and were grown in a chamber at 30 °C and light intensity of 150–170 μmol photons m^−2^ s^−1^. When the cotyledons were fully expanded, the Jatropha seedlings were transferred to a hydroponic container of 100 mm diameter and 110 mm height, which was filled with 800 mL of half-strength Hoagland’s solution (2 mM C_6_H_13_NO_4_S, 2.5 mM KNO_3_, 2.5 mM Ca(NO_3_)_2_, 1 mM MgSO_4_, 0.5 mM KH_2_PO_4_, 6 µM Fe(III)-EDTA, 4 µM H_3_BO_3_, 1.3 µM MnCl_2_, 87 nM ZnSO_4_, 40 nM CuSO_4_, 20 nM Na_2_MoO_4_). The pH of the solution was adjusted to 5.5 with KOH. Hydroponic culture was carried out in a culture room set at 25 °C, 14 h/10 h light/dark cycle, and a light intensity of 150–170 μmol photons m^−2^ s^−1^. The hydroponic solution was aerated at a rate of 45 mL min^−1^ using an air pump. The hydroponic solution was changed 7 days after the start of hydroponic culture and then changed every 3 days thereafter. When the first true leaf was fully developed, the hydroponic solution was changed to new half-strength Hoagland’s solution containing either CdSO_4_, Cr_2_(SO_4_)_3_, CuSO_4_, NiSO_4_, or ZnSO_4_ at concentrations of 1, 10, or 100 µM for each PTE. The PTE-containing hydroponic solutions were changed at 3-day intervals up to day 12. Plants grown in hydroponic solution without the addition of PTEs were used as controls.

### Growth and physiological measurements

SPAD values^13^ of the first and second leaves of Jatropha seedlings were measured using a SPAD-502Plus chlorophyll meter (Konica Minolta, Tokyo, Japan) 12 days after introducing the PTEs. SPAD values were measured at three different points on each leaf, using a Reading Checker Plate (Konica Minolta) for quality control, and the values were averaged. For the measurement of dry weight, Jatropha plants were oven-dried at 70 °C for approximately 1 week, and the remaining dry matter of the shoot and root tissues was weighed using an analytical balance. Photosynthetic CO_2_ fixation rate and ETR were measured from the second leaf using an LI-6400 portable photosynthesis system (LI-COR, Lincoln, NE) at 25 °C. CO_2_ flow rate was set at 400 μmol mol^−1^, and photosynthetically active radiation intensity of 50, 150, 300, and 600 μmol photons m^−2^ s^−1^ were illuminated during the analysis. Dark-adapted *F*_*v*_*/F*_*m*_ ratio was measured using a miniPAM (Heinz Walz GmbH, Bayern, Germany) after dark adaptation for 1 h, and calculated as described previously^46^. Water content of the leaf and root tissues were calculated from their fresh weight (FW) and DW using the following equation (Water content (%) = (FW — DW)/FW × 100). Transpiration rate of the fully expanded second leaves was measured using a Leaf Porometer OSC-1 (Decagon Devices, Pullman, WA).

### Measurements of PTEs and mineral nutrients accumulation

After hydroponic culturing for 12 days, plants were transferred to a vessel filled with distilled water and their roots were briefly rinsed. The harvested roots were washed with 100 mL of 1 M HCl solution for 1 min, washed with a solution of 0.1 M Na_2_ EDTA, and finally rinsed with distilled water to remove the applied metals adhering to the surface^47^. After oven-drying for the weight measurements as described above, the dried tissues were homogenized using a homogenizer Shake Master (Biomedical Sciences, Tokyo, Japan), and coarse particles were ground into a fine powder using a mortar and pestle.

Nitrate decomposition was carried out using the method of Munter *et al*.^48^, with the following modification. One gram of dry sample was dissolved in 10 mL of concentrated HNO_3_ in a flask and was digested for 45 min on a hot plate at 90 °C. The solution was evaporated at 140 °C until the volume of the solution was reduced to approximately 1 mL. The solution was allowed to cool to room temperature and then increased to 25 mL with 1% HNO_3_. Contents of PTEs and mineral nutrients in the decomposed samples were measured using inductively coupled plasma atomic emission spectroscopy (SPECTRO CIROS CCD, SPECTRO Analytical Instruments GmbH, Nordrhein-Westfalen, Germany), using the Metal Standard Solutions (registered by the Japan Calibration Service System, Wako Chemicals, Osaka, Japan) as reference materials for quality and quantity assurance. EFs of the minerals were calculated using the following equation: EF = metal concentration in the plant tissue/metal concentration in the hydroponic solution.

### Determination of thiol compounds

Quantification of thiol compounds in the tissues was performed by the Ellman reaction^49^ in accordance with the method of Rascio *et al*.^50^. Leaf (approximately 4 cm^2^) and root (approximately 0.2 g) samples of Jatropha tissues were weighed and then ground into powder in liquid nitrogen with a mortar and pestle. Then, 15 mL of homogenization solution containing 20 mM Tris-HCl (pH 8) and 100 mM sodium ascorbate was added to the powder, and it was homogenized with a mortar and pestle again. The extracts were filtered through Miracloth (Merck KGaA, Darmstadt, Germany), the filtrates centrifuged at 10,000 × *g* for 25 min at 4 °C, and the supernatant transferred to a new tube. The Ellman reaction mixture (800 μL) was composed of 20 mM sodium phosphate buffer (pH 8.0), 200 μL of the extract, and 20 μL of 10 mM 5,5′-dithio-bis-[2-nitrobenzoic acid] (DTNB) solution. The reaction was started by adding the DTNB solution, and samples were incubated at room temperature for 1 h in the dark. Absorbance of the reaction mixture was measured at 412 nm, and moles of SH compounds were calculated based on the molecular coefficient of the SH groups for the Ellman reaction (14,150 M^−1^) as described previously^51^.

## Electronic supplementary material

Supplementary Information
