# Supplementary material for: Differential physiological responses and tolerance to potentially toxic elements in biodiesel tree Jatropha curcas
Source: Sci Rep. 2018 Jan 26;8:1635. doi: 10.1038/s41598-018-20188-5 (PMC5786012; doi:10.1038/s41598-018-20188-5)
Supplement: Supplementary file 1 — Supplementary Information [file 41598_2018_20188_MOESM1_ESM.pdf]

Supplemental Tables

Differential physiological responses and tolerance to potentially toxic elements in biodiesel tree *Jatropha curcas*

Minami Yamada[1], Goitseone Malambane[2], Satoshi Yamada[1,2], Sony Suharsono[3], Hisashi Tsujimoto[2,4], Baleseng Moseki[5], Kinya Akashi[1,2,4*]

[1]Graduate School of Agriculture Study, Tottori University, 4-101 Koyama-Minami, Tottori 680-8553, Japan

[2]The United Graduate School of Agricultural Sciences, Tottori University, 4-101, Koyama-minami, Tottori 680-8553, Japan

[3]Research Center for Bioresources and Biotechnology, Bogor Agricultural University, Gd. PAU, Kampus IPB Darmaga, Bogor 16680, Indonesia

[4]Arid Land Research Center, Tottori University, 1390 Hamasaka, Tottori 680-0001, Japan

[5]Department of Biological Sciences, University of Botswana, Private Bag UB 00704, Gaborone, Botswana

*Corresponding author. Email: akashi.kinya@muses.tottori-u.ac.jp

Supplementary Table S1. PTE content in the HCl washing solution of Jatropha roots

Treatment[*1]	PTE content in the wash solution[*2] (nmol metal/plant)				
	Cd	Cr	Cu	Ni	Zn
Control	5.1	14.2	115.5	1.8	76.2
100 µM Cd	7129	n.d.	144	3	37.5
100 µM Cr	n.d.	8748.1	117.5	9.1	38.2
100 µM Ni	n.d.	n.d.	59.4	357.5	10.8

[*1] PTE and its concentration applied to the hydroponic solution.
[*2] Jatropha roots were washed with 100 mL of 1 N HCl, and metal concents in the HCl solution were measured.
n.d., not detected (detection limit >1 nmol metal/plant).

Supplementary Table S2. Accumulation of PTEs in Jatropha seedlings grown in the metal-supplemented hydroponic culture

Additive	Concentration (μM)	PTE content in the plant (nmol metal gDW^{-1})				
		Cd	Cr	Cu	Ni	Zn
none (control)		0.6 ± 0.8 a	160.2 ± 140.0 a	50.5 ± 21.8 a	8.2 ± 7.5 a	227.0 ± 141.5 a
Cd	1	676.0 ± 129.7 a	169.9 ± 135.7 a	56.8 ± 17.9 a	4.1 ± 3.9 a	255.0 ± 51.0 a
	10	2796.5 ± 1044.3 b	66.7 ± 19.9 a	70.9 ± 32.3 a	8.7 ± 5.8 a	209.6 ± 57.1 a
	100	7647.6 ± 469.6 c	340.4 ± 206.8 a	146.7 ± 24.6 a	138.4 ± 165.4 a	459.0 ± 119.1 a
Cr	1	0.2 ± 0.4 a	617.9 ± 550.7 a	35.1 ± 10.5 a	6.2 ± 5.9 a	129.6 ± 34.7 a
	10	0.6 ± 1.1 a	5339.9 ± 1751.9 b	41.7 ± 17.5 a	12.4 ± 8.0 a	150.5 ± 64.4 a
	100	1.8 ± 1.1 a	31482.2 ± 4270.5 c	66.5 ± 6.8 a	16.8 ± 17.7 a	225.8 ± 51.2 a
Cu	1	2.8 ± 1.5 a	117.5 ± 133.6 a	88.2 ± 25.6 a	4.3 ± 7.5 a	294.8 ± 62.1 a
	10	27.6 ± 45.5 a	46.6 ± 52.7 a	383.3 ± 98.5 a	5.8 ± 10.0 a	309.0 ± 45.5 a
	100	2.5 ± 1.1 a	374.4 ± 158.5 a	3718.7 ± 905.6 b	50.3 ± 49.2 a	518.0 ± 59.5 a
Ni	1	2.0 ± 0.5 a	270.5 ± 363.2 a	60.4 ± 2.0 a	146.4 ± 41.0 a	277.4 ± 26.0 a
	10	1.2 ± 0.4 a	20.8 ± 19.8 a	44.7 ± 16.3 a	556.3 ± 175.5 a	166.6 ± 67.0 a
	100	3.6 ± 4.1 a	218.6 ± 260.8 a	134.8 ± 24.7 a	1859.7 ± 1027.7 b	495.5 ± 89.0 a
Zn	1	3.0 ± 1.7 a	154.6 ± 184.6 a	77.1 ± 1.0 a	9.3 ± 9.1 a	562.1 ± 50.2 a
	10	1.9 ± 0.4 a	134.4 ± 143.6 a	96.7 ± 11.8 a	8.0 ± 8.3 a	3564.7 ± 803.3 ab
	100	0.9 ± 1.5 a	63.6 ± 55.1 a	48.3 ± 45.6 a	8.4 ± 8.0 a	9668.7 ± 9753.3 b

Values are expressed as the average ± standard deviation (n = 3)
Different alphabets indicate significant difference by Tukey-Kramer test (a<b<c, P<0.05).

Supplementary Table S3. Enrichment factors of PTEs in Jatropha

Additive	Conc. (μM)[*1]	Enrichment factor[*2]
Cd	1	87.3 ± 16.7
	10	36.1 ± 13.4
	100	9.8 ± 0.6
Cr	1	79.8 ± 71.1
	10	68.9 ± 22.6
	100	40.6 ± 5.5
Cu	1	11.3 ± 3.3
	10	4.9 ± 1.2
	100	4.8 ± 1.1
Ni	1	18.9 ± 5.2
	10	7.1 ± 2.2
	100	2.4 ± 1.3
Zn	1	72.5 ± 6.4
	10	46.0 ± 10.3
	100	12.4 ± 12.5

[*1] Concentration in the hydroponic solution.
[*2] Values are expressed as the average ± standard deviation (n = 3).

Supplementary Table S4. Distribution of PTEs in the shoots and roots of Jatropha treated with 10 µM PTE

Measured metal	Treatment[*1]	Tissue	Metal content (nmol metal gDW^{-1}) [*2]
Cd	Control	Shoot	3.5 ± 0.9
		Root	31.6 ± 32.9
	Cd	Shoot	1392.0 ± 510.2
		Root	14488.9 ± 1841.4
Cr	Control	Shoot	76.2 ± 12.9
		Root	255.3 ± 288.2
	Cr	Shoot	164.6 ± 82.3
		Root	36146.4 ± 4867.7
Cu	Control	Shoot	72.1 ± 33.4
		Root	74.5 ± 13.9
	Cu	Shoot	163.0 ± 24.8
		Root	2122.7 ± 644.0
Ni	Control	Shoot	15.0 ± 1.8
		Root	28.9 ± 11.7
	Ni	Shoot	498.5 ± 176.9
		Root	3769.4 ± 233.9
Zn	Control	Shoot	189.7 ± 65.2
		Root	170.0 ± 31.1
	Zn	Shoot	1441.3 ± 220.6
		Root	12860.4 ± 3825.0

[*1] Concentration of respective PTEs in the hydroponic solution was set at 10 µM.

[*2] Values are expressed as the average ± standard deviation (n = 3)
